# Association between diabetes mellitus and primary biliary cholangitis: a two-sample Mendelian randomization study

**DOI:** 10.3389/fendo.2024.1362584

**Published:** 2024-05-07

**Authors:** Dan Lv, Han Wang, Yan Leng, Sitong Chen, Haitao Sun, Xiangyue Meng, Tiejun Liu, Zhuang Xiong

**Affiliations:** ^1^College of Traditional Chinese Medicine, Changchun University of Chinese Medicine, Changchun, China; ^2^Department of Hepatology, First Affiliated Hospital to Changchun University of Chinese Medicine, Changchun, China; ^3^College of Integrative Medicine, Changchun University of Chinese Medicine, Changchun, China

**Keywords:** primary biliary cholangitis, diabetes mellitus, type 1 diabetes, type 2 diabetes, Mendelian randomization, causality

## Abstract

**Background:**

Previous observational studies have demonstrated a link between diabetes mellitus(DM) and primary biliary cholangitis (PBC). Nevertheless, since these relationships might be confused, whether there is any causal connection or in which direction it exists is unclear. Our investigation aimed to identify the causal associations between DM and PBC.

**Methods:**

We acquired genome-wide association study (GWAS) datasets for PBC, Type 1 diabetes(T1DM), and Type 2 diabetes(T2DM) from published GWASs. Inverse variance-weighted (IVW), MR-Egger, weighted median (WM), Simple mode, and weighted mode methods were used to determine the causal relationships between DM(T1DM or T2DM) and PBC. Sensitivity analyses were also carried out to ensure the results were robust. To determine the causal relationship between PBC and DM(T1DM or T2DM), we also used reverse MR analysis.

**Results:**

T1DM was associated with a higher risk of PBC (OR 1.1525; 95% CI 1.0612-1.2517; *p* = 0.0007) in the IVW method, but no evidence of a causal effect T2DM on PBC was found (OR 0.9905; 95% CI 0.8446-1.1616; *p* = 0.9071) in IVW. Results of the reverse MR analysis suggested genetic susceptibility that PBC was associated with an increased risk of T1DM (IVW: OR 1.1991; 95% CI 1.12-1.2838; *p* = 1.81E-07), but no evidence of a causal effect PBC on T2DM was found (IVW: OR 1.0101; 95% CI 0.9892-1.0315; *p* = 0.3420).

**Conclusion:**

The current study indicated that T1DM increased the risk of developing PBC and vice versa. There was no proof of a causal connection between PBC probability and T2DM. Our results require confirmation through additional replication in larger populations.

## 1 Introduction

Primary biliary cholangitis (PBC) is an autoimmune disease characterized by positive serum antimitochondrial antibodies (AMA), elevated alkaline phosphatase (ALP), and histological manifestations of non-suppurative destructive cholangitis, which ultimately leads to hepatic fibrosis as well as cirrhosis and hepatocellular carcinoma, and the pathogenesis of which is yet to be fully defined (1). Statistically, the prevalence of PBC per 100,000 people in Europe, North America, and the Asia-Pacific region ranges from 1.91 to 40.2 (2). Ursodeoxycholic acid (UDCA) is globally recognized as the most effective therapy for PBC because of its ability to improve patients’ biochemical indexes, alleviate pathological changes, and decrease disease development; however, up to 40% of patients do not respond to UDCA treatment (3). PBC has been linked to several extrahepatic immune-mediated disorders in the past few years, including DM, celiac disease, inflammatory bowel disease, and rheumatoid arthritis (4–7).

The incidence of DM is increasing globally, with the projected global incidence of DM among individuals aged 20-79 years estimated to reach 12.2% (783.2 million individuals) by 2045. This surge poses a significant menace to individuals’ health and well-being (8). Diabetes has a long history of causing liver damage. In a retrospective cohort study in China, T2DM was found to be one of the major metabolic risk factors for PBC, with 11.9% of patients with T2DM having lower albumin, platelet counts and a higher rate of cirrhosis than non-T2DM patients; however, 54.3% of these patients also had hyperlipidemia, hypertension and NAFLD (9), which caused some interference in the study. In the most recent years, several epidemiologic studies have demonstrated a strong correlation between DM and autoimmune liver disease. A case-control study including 36,467 patients with AIH, 39,924 patients with PBC, and 4,877 patients with PSC showed an incidence of T1 DM of 1.7% and T2 DM of 18.1% in patients with PBC (10), suggesting that raising awareness of the risk of diabetes in patients with autoimmune liver disease is necessary. In another study, DM was strongly associated with the progression of PBC. Six non-invasive scores (FIB-4, APRI, RPR, MRS, the Newcastle model, and ALBI scores) were used to predict the severity of hepatic fibrosis, and it was found that non-invasive scores of PBC-DM were significantly higher than those of PBC patients. Effective management of DM could slow down the progression of PBC to cirrhosis, as it was observed that the occurrence of cirrhosis was notably higher in patients (62.2%) when compared to those with PBC alone (42%) (7). It can be seen that improving the treatment and monitoring of DM in patients with PBC is one of the most essential tools to prevent disease progression (11). These findings suggest a robust clinical association between PBC and DM. However, there are limited studies on the potential causal relationship and pathologic mechanisms between DM and PBC, and further studies are needed to confirm this.

The ability of traditional observational studies to infer causality is vulnerable to potential confounding and reverse causation. An epidemiological methodology called MR analysis can support causal inference by employing genetic variations as instrumental variables (IVs) for exposure (12).MR analysis is unique compared to observational studies. It reduces confounding bias and prevents reverse causation because genetic alleles are randomized at conception, and disease cannot change germline genotype (13). As a result, MR has been applied more frequently to evaluate likely causal links between exposures and results (14). MR analysis uses genetic variation as IVs to detect and quantify causal relationships (15). The MR research method, which uses single nucleotide polymorphisms (SNPs) as IVs, is unaffected by environmental factors and can effectively control the interference of confounding factors, similar to randomized trials. The present study implemented a two-sample bidirectional MR study to investigate the potential causality between two main subtypes of DM(T1DM and T2DM) and PBC outcomes using large-scale GWAS data.

## 2 Materials and methods

### 2.1 Study design

**Figure 1:**
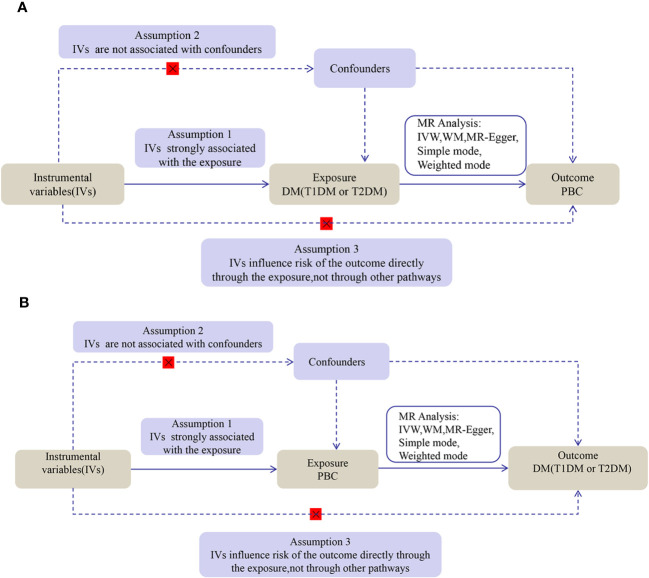
The flow diagram of the MR analysis. **(A)** DM SNPs were employed as the genetic instruments to examine the causal effect of DM on PBC. **(B)** PBC SNPs were employed as the genetic instruments to examine the causal effect of PBC upon DM. DM, diabetes mellitus; T1DM, Type 1 diabetes; T2DM, Type 2 diabetes; PBC, primary biliary cholangitis; MR, Mendelian randomization; IVW, inverse variance weighted; WM, weighted median.

We performed a two-sample bidirectional MR study using openly published GWAS summarized data. The MR analysis proceeded on the basis of three key assumptions: (1) IV must be strictly related to DM (T1DM and T2DM); (2) IV must be independent of confounders between DM (T1DM and T2DM) and PBC; and (3) IV will not affect PBC due to factors besides DM (T1DM and T2DM). The first analysis investigated causality between DM (T1DM and T2DM) as an exposure and PBC as the outcome, and the second analysis investigated reverse causality, with PBC as the exposure factor and DM (T1DM and T2DM) as the outcome. The flow for this MR study is shown in **Figure 1**.

### 2.2 Data sources

All summary statistics were obtained from the IEU OpenGWAS database (https://gwas.mrcieu.ac.uk/). The T1DM GWAS included 2,649 cases and 183,674 controls(https://gwas.mrcieu.ac.uk/datasets/finn-b-E4_DM1_STRICT/), while the T2DM GWAS included 29,166 cases and 183,185 controls (https://gwas.mrcieu.ac.uk/datasets/finn-b-E4_DM2_STRICT/). The T1DM and T2DM cohort populations were of European descent. Meanwhile, the PBC GWAS dataset included 2764 cases and 10475 controls of European descent. (https://gwas.mrcieu.ac.uk/datasets/ebi-a-GCST003129/ The PBC cases within the cohort fulfilled the criteria the American Association for the Study of Liver Diseases set forth for PBC (16). To mitigate population stratification bias, we exclusively utilized data from studies that specifically focused on populations of European origin. No additional ethical approval was necessary as all the data were publicly available.

### 2.3 Selection of IVs

In this study, we selected single nucleotide polymorphisms (SNPs) closely associated with T1DM (*p* < 5 × 10 ^- 6^) and also identified SNPs closely related to T2DM and PBC (*p* < 5 × 10 ^- 8^). In order to guarantee the independence of SNPs, we eliminated those with linkage disequilibrium (r^2 =^ 0.001, genetic distance = 10,000 kb). To mitigate the influence of IVs on the causal analysis, we incorporated the F statistic, which indicated a value higher than 10. The computation of the F statistic followed the formula: F = (R^2^/(1 - R^2^)) * ((n - k - 1)/k) (17); here, n represents the sample size, and R2 denotes the variance explained by the IVs. The calculation of R2 involved the minor allele frequency (MAF) and the value of β, as follows: R^2 =^ 2 * MAF * (1 - MAF) * β^2^. We then extracted the remaining SNPs from the ending summary statistics. SNPs significantly associated with the outcomes directly were dropped to meet the third assumption. After harmonizing SNPs-exposure and SNPs-outcome, we excluded SNPs in palindromes based on allele frequencies, while to avoid potential pleiotropy, we used PhenoScanner V2 (https://www.phenoscanner.medschl.cam.ac.uk/) to exclude association with outcome confounders or risk factors of IVs (18). To ensure the reliability of MR estimates (19), we conducted MR-pleiotropy residual sum and outlier (MR-PRESSO) analysis before the MR analysis. This analysis helped identify and remove any outliers with potential pleiotropy. The SNPs that remained after these analyses were then used as genetic instruments, following the abovementioned steps.

### 2.4 Estimation of causal effect

After compiling the list of SNPs based on the selection above criteria, we performed a forward MR analysis to assess the total impact of these selected SNPs for DM on PBC. IVW, simple mode, weighted mode, MR-Egger regression, and WM were all used in this investigation. The validity of the results was verified by comparing the effect estimates obtained from the five different MR methods since the horizontal pleiotropy of the IVs could skew the results. The identical MR methods were then applied to the reverse-direction MR analysis. The odds ratios (OR) for effect estimates were given with 95% confidence intervals (CI).

### 2.5 Sensitivity analysis

We employed the MR-Egger intercept test to determine whether horizontal pleiotropy was present. The results should be regarded cautiously if the intercept is significant (*p* < 0.05). The MR-Egger intercept test findings were displayed using scatter plots. Additionally, we looked at heterogeneity using Cochran’s Q statistics, where substantial heterogeneity (*p* < 0.05) denotes the existence of heterogeneity among the included studies. Funnel plots were used to show the results. We apply the MR-PRESSO outlier test to exclude aberrant SNPs (outliers) and estimate the adjusted values to eliminate horizontal pleiotropy. In the leave-one-out study, forest plots were created to visually assess the robustness of the results after SNPs were removed one at a time.

### 2.6 Statistical analysis

The MR-PRESSO (version 1.0) and TwoSampleMR (version 0.5.7) packages in R Version 4.3.1 were used for these MR analyses. Moreover, the ggplot2 package produced graphs. The sensitivity and MR analysis results about the exposures and outcomes were deemed statistically significant at *p* < 0.05 after both sides ran their statistical tests.

## 3 Results

### 3.1 The causal effect of T1DM and T2DM on PBC

#### 3.1.1 Effect of T1DM on PBC

This study obtained 11 SNPs associated with T1DM, which met the universally accepted genome-wide significance threshold (*p* < 5 × 10^−6^, r^2^ = 0.001, distance = 10,000 kb) for exposure. One SNP (rs8029659) in T1DM was removed to eliminate smoking-related confounding factors. Another SNP (rs11203203) related to PBC-relevant trait was ruled out. Additionally, our MR analysis demonstrated no instances where we utilized a feeble instrument (all F-statistics>10). Finally, the remaining 9 SNPs were selected as IVs for T1DM (**Supplementary Table 1**).

A strong association was discovered between T1DM and PBC (IVW: OR 1.1525; 95% CI 1.0612-1.2517; *p* = 0.0007). WM (WM: OR1.1513; 95% CI 1.0895-1.2166; *p* = 5.61E-07), Simple mode (Simple mode: OR 1.29; 95% CI 1.1178-1.4888;*p* = 0.0082)and weighted mode (weighted mode: OR 1.1520; 95% CI 1.0917-1.2157;*p* = 0.0008) confirmed the T1DM - PBC association. MR-Egger regression showed a consistent direction but insignificant result (OR 1.1554,95%CI 1.0191-1.3099, *p* = 0.0587) (**Supplementary Table 4**).

The analysis of T1DM on PBC showed significant heterogeneity according to Cochran’s Q test (Q = 22.292; *p* = 0.0044). However, the observed heterogeneity in some outcomes did not undermine the MR estimates since the random-effect IVW adopted in this study was able to mitigate the pooled heterogeneity. Furthermore, the p-value was greater than 0.05 in the MR-PRESSO global tests, indicating the absence of horizontal pleiotropy across the analyses. (**Supplementary Table 6**).

#### 3.1.2 Effect of T2DM on PBC

Based on the abovementioned procedures and criteria, 24 SNPs (*p* < 5 × 10 ^- 8^)were tentatively chosen as IVs for T2DM. Removing the following SNPs for being palindromic with intermediate allele frequencies: rs11712037, rs6780171, and rs745805, finally, 21 SNPs were screened as genetic instruments for T2DM (**Supplementary Table 2**). After analysis, we found no evidence of a causal effect T2DM on PBC (IVW: OR 0.9905; 95% CI 0.8446-1.1616; *p* = 0.9071), WM (WM: OR 0.8710; 95% CI 0.7139-1.0627; *p* = 0.1736), and MR Egger (MR Egger: OR 0.8415; 95% CI 0.6148-1.1518; *p*= 0.2948) confirmed these findings (**Supplementary Table 4**). No considerable or suggestive correlation was found between the genetic predisposition for T2DM and the likelihood of PBC (*p*> 0.05). Therefore, additional examination of heterogeneity and pleiotropy was unnecessary. However, it is important to note that our statistical power might have been insufficient to identify these tenuous connections.

### 3.2 The causal effect of PBC on T1DM and T2DM

#### 3.2.1 Effect of PBC on T1DM

Furthermore, we performed an MR analysis in the opposite direction, exploring the relationship between PBC and DM subtypes. Throughout this reverse-direction MR analysis, 24 SNPs were provisionally identified as IVs for PBC, exhibiting significance at the genome-wide threshold (*p* < 5 × 10^-8^) and demonstrating independent inheritance (r^2^ = 0.001 and distance = 10,000 kb) from the pool of 1,124,241 SNPs. Two SNPs(rs12924729 and rs2304256)associated with T1DM-relevant traits were found by inquiring about the PhenoScanner V2 database. Six SNPs (rs10488631 rs485499 rs4938573 rs6679356 rs7774434 rs9591325) were excluded from the analysis because of being outliers identified by MR-PRESSO. In the end, MR analysis accepted 16 SNPs to evaluate the causal impact of PBC on T1DM (**Supplementary Table 3**). Assessing the appropriateness of genetic instruments for MR analysis, they proved suitable based on F statistics and the proportion of variance explained (R^2^).

A strong association was discovered between PBC and T1DM (IVW: OR 1.1991; 95% CI 1.12-1.2838; *p* = 1.8E-07). WM (WM: OR 1.1947; 95% CI 1.0932-1.3056; *p* = 0.0001) and weighted mode (weighted mode: OR 1.2095; 95% CI 1.0669-1.3711; *p*= 0.0087) confirmed the PBC and T1DM association (**Supplementary Table 5**).

Cochran’s Q test revealed no significant heterogeneity in the analysis of total PBC on T1DM(Q = 16.3371; *p* = 0.3600). No directional pleiotropy bias was found in the MR-Egger test (Intercept = 0.0061; *p* = 0.8640). No SNP outliers were found in the MR-PRESSO global test (RSSobs = 18.0021; *p* = 0.4364) and leave-one-out MR analysis (**Supplementary Table 6**).

#### 3.2.2 Effect of PBC on T2DM

In the same way, we also performed a reverse magnetic resonance investigation between T2DM and PBC. However, the IVW approach did not uncover any reverse causal associations (IVW: OR 1.0101; 95% CI 0.9892-1.0315; *p* = 0.3420). Consistent results were obtained using WM, Simple mode, and Weighted mode. The findings from the reverse MR analysis can be found in **Supplementary Table 5**. Consequently, there was no need for additional examination of heterogeneity and pleiotropy.

**Figure 2:**
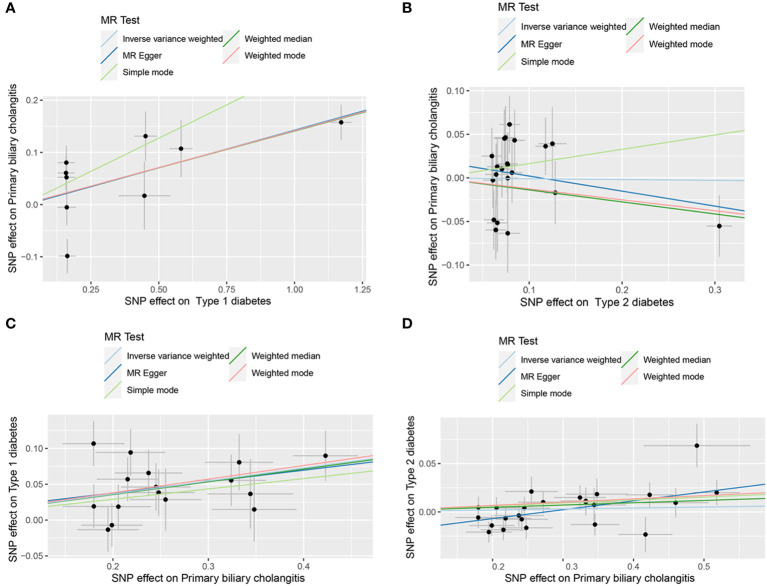
Scatter plots of primary MR analysis. **(A)** T1DM on PBC; **(B)** T2DM on PBC; **(C)** PBC on T1DM; **(D)** PBC on T2DM. T1DM, Type 1 diabetes; T2DM, Type 2 diabetes; PBC, primary biliary cholangitis; MR, Mendelian randomization; IVW, inverse variance weighted; WM, weighted median.

**Figure 3:**
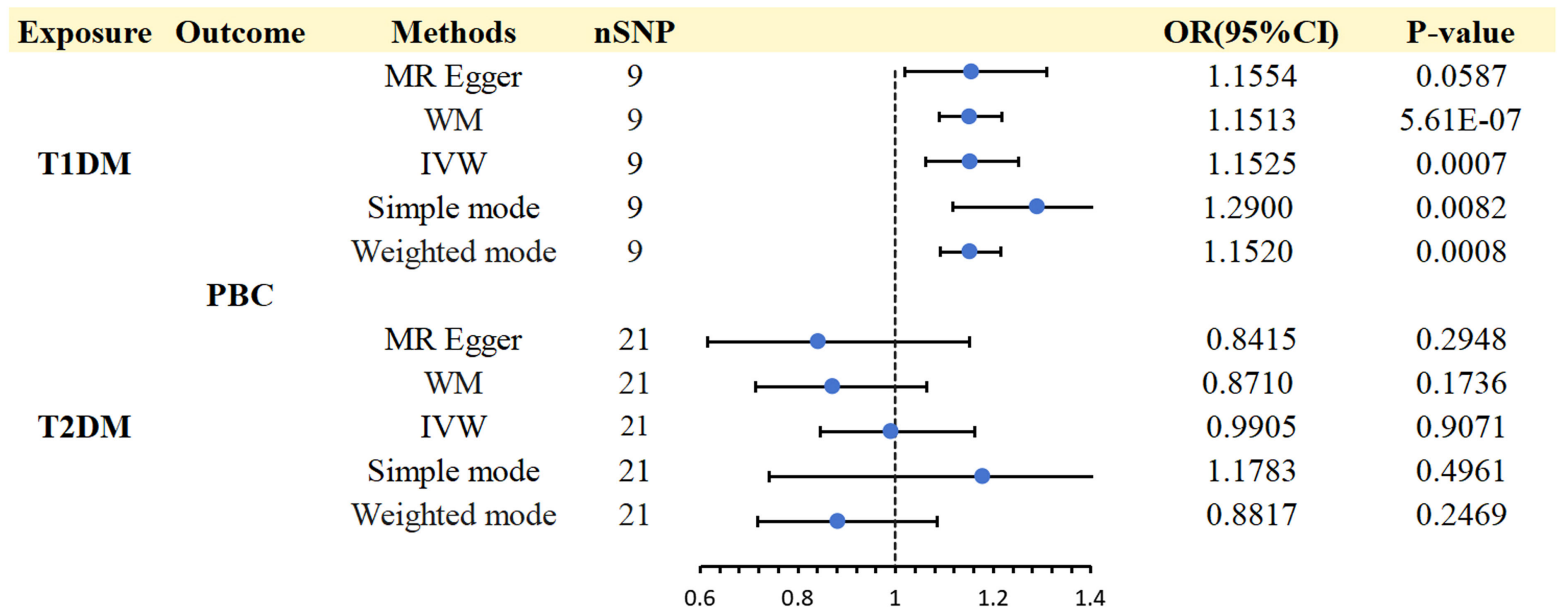
Causal effects for DM (T1DM OR T2DM) on PBC.

**Figure 4:**
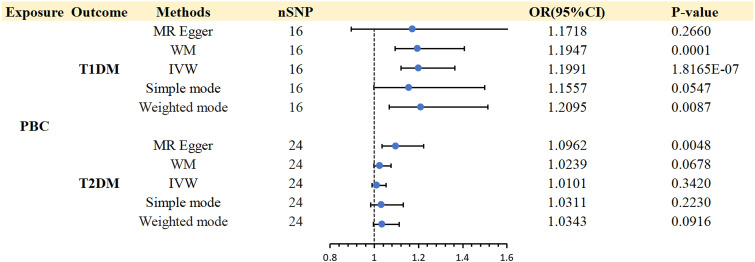
Causal effects for PBC on DM (T1DM OR T2DM).

The scatter plots of causal relationships of MR analyses are shown in **Figure 2**. The causal relationships are shown in **Figures 3** and **4**. The funnel, leave-one-out and forest plots of MR analyses are shown in **Supplementary Figures 1–3**.

## 4 Discussion

PBC is an autoimmune liver disease of which pathogenesis is still unclear, and the clinical treatment effect is unsatisfactory. PBC’s biochemical insensitivity to UDCA and the development of liver cirrhosis or fibrosis put it at a markedly higher risk of progressing to hepatocellular carcinoma (20). According to the results of a long-term follow-up study on 1,615 patients with early-stage PBC, it was found that 50% of the patients progressed to a more severe stage within five years. This information can help healthcare professionals identify those at higher risk and provide early intervention to prevent or delay disease progression (21). Investigating diseases that may increase the risk of developing PBC cannot be overlooked. This critical step ensures early detection and effective prevention strategies. Megyesi et al. proposed the concept of Hepatic Diabetes (HD) in 1967, pointing out that patients with chronic liver disease are more likely to have impaired glucose tolerance and complicated diabetes (22). Although some researchers have studied the relationship between DM and liver disease, the majority of studies have solely focused on non-alcoholic fatty liver disease (23, 24), and little information is available on the potential relationship between DM and PBC risk. Meanwhile, previous findings in the literature were limited to observing correlations, and reverse causality may not be avoided. By leveraging the power of MR, we delved into the correlation between DM and PBC risk, employing a vast array of genomic data. Our findings present a compelling method for investigating this crucial link. We selected two main DM subtypes (T1DM and T2DM) with sufficient sample size. Investigating the relationship between PBC and T1DM or T2DM susceptibility utilized multiple MR methods and reverse MR. This approach allowed for a comprehensive and in-depth analysis, providing valuable insights into the underlying mechanisms. It is intriguing that genetically determined T1DM has a suggestive correlation with an increased risk of PBC. The pattern of association between genetically determined T2DM and PBC risk was unclear. Reverse MR Analysis found that patients with PBC were causally associated with increased risks of T1DM, but no causal relationship between PBC and T2DM. This groundbreaking MR study has provided the first-ever estimation of the causal relationship between DM and PBC, with sensitivity tests verifying that outliers, horizontal pleiotropy, and reverse causality were not contributing factors.

However, the mechanisms underlying this association are unidentified. As one of the chronic autoimmune diseases, T1DM is characterized by pancreatic beta cell destruction or damage leading to insulin deficiency and hyperglycemia (25). Over the years, the incidence and prevalence of T1DM have been on the rise, bringing a severe economic burden to patients’ families and lives (26–28). T1DM and PBC are both autoimmune diseases involving interactions between genetic and immune factors, raising the possibility that they are somehow related. Observations of diverse autoimmune diseases have been frequently reported (29, 30). Although there is insufficient biochemical or genetic evidence, the idea or hypothesis of shared autoimmunity has been established to recognize these concurrencies. Through analysis, it was predicted that T1DM could be predicted from the high expression of 7 pivot genes: DNA Damage Inducible Transcript 4 (DDIT4), Establishment Of Sister Chromatid Cohesion N-Acetyltransferase 2 (ESCO2), SH3 Domain Binding Protein 4 (SH3BP4), Prickle Planar Cell Polarity Protein 1 (PRICKLE1), EPM2A Interacting Protein 1 (EPM2AIP1), Potassium Inwardly Rectifying Channel Subfamily J Member 15 (KCNJ15) and Glutamate Metabotropic Receptor 8 (GRM8). According to genome enrichment analysis (GSEA), most of these central genes may be primarily in alterations such as inflammation, infection, immunity, cancer, and apoptosis. At the same time, the exposure levels of these central genes have also changed in several other autoimmune diseases, including PBC, suggesting that they may be common targets in these autoimmune diseases (31). T1DM arises from a somatic mutation occurring in the epitope-binding groove of an HLA gene that is predisposed to risk. This mutation directly impacts the binding affinity between the HLA-insulin-peptide-TCR complex, thereby triggering an autoimmune pathway. The specific autoimmune disease that manifests is contingent upon the peptide that binds to the mutated epitope-binding groove of the HLA gene. This connection also gives rise to the potential occurrence of multiple autoimmune diseases stemming from a single at-risk HLA locus. Consequently, T1DM and common autoimmune diseases exhibit a comparable etiology centred around somatic mutations (32). In animal experiments, researchers utilized a traditional method of recombining breeding to exhibit that the NOD background’s existence of the altered Pkhd1del36-67 prompts the emergence of autoimmune biliary disease, showcasing resemblances to human PBC (33). It is interesting to note that reports of T1DM and PBC development in humans have also been made (34). The occurrence rate of primary biliary cholangitis (PBC) is considerably higher in women compared to men; nonetheless, a case report highlights an atypical occurrence of PBC in a male individual diagnosed with T1DM (35). These echo the results of our study - T1DM and PBC may be causally related.

Although some studies have shown that T2DM and PBC often co-exist (10, 36, 37), our investigation utilizing MR analysis did not reveal any substantiating proof for a causal influence of T2DM on PBC, and vice versa. PBC causes minor bile duct epithelial cell injury, cholestasis, and immune regulation disorders but also causes severe metabolic abnormalities, especially glucose metabolism (38). In a retrospective examination of the medical documents on individuals diagnosed with PBC, the study gathered follow-up information by conducting periodic, standardized telephone interviews. The results unveiled a higher prevalence of type 2 diabetes and greater liver ailment severity at the baseline among patients afflicted with gallstone disease (39). A case report demonstrated that hepatic inflammation plays a vital role in the pathogenesis of T2DM by systemic insulin resistance in chronic liver disease, including PBC (40). Abnormal bile acid metabolism is one of the essential mechanisms of PBC. At the same time, bile acids also play an important role in glucose metabolism (41). The reduced expression of farnesoid X receptor (FXR) in PBC patients limits the role of bile acids, leading to insulin resistance and thus affecting glucose metabolism (42). Previous studies have demonstrated that FXR agonists improve hyperglycemia and hyperlipidemia in diabetic mice by inhibiting liver gluconeogenesis and enhancing insulin sensitivity by increasing liver glycogen synthesis and glycogen content (42, 43). Nonetheless, another study showed that FXR antagonists inhibit hepatic gluconeogenesis through the FXR/miR-22-3p/PI3K/AKT/FoxO1 pathway and promote glycogen synthesis via the FXR/miR-22-3p/PI3K/AKT/GSK3β pathway, thereby improving glucose homeostasis in T2DM mice (44).In addition, it is currently believed that defects in Anion exchanger-2 (AE2), a Cl-/HCO3- exchanger located in the apical membrane of the BEC that pumps HCO3- out of the cell, is one of the main contributors to changes in bile acid metabolism. Biliary HCO3 secretion will prevent bile acids from invading bile duct cells and inducing cytotoxicity. Studies have shown that reduced levels of AE2 mRNA and AE2-associated dysfunction in liver and peripheral blood mononuclear cell specimens from patients with PBC may play a role in the pathogenesis of PBC (45, 46). Paradoxically, another study showed that AE2 may be a glucose-sensitive transmembrane transporter and a new potential therapeutic target for diabetic vasculopathy. High glucose can upregulate the expression and activity of AE2, increase [Cl(-)]i in a time- and concentration-dependent manner, induce cell apoptosis, and produce diabetic vasculopathy (47). In addition, it is now widely accepted that the development of oxidative stress, lipotoxicity, and endoplasmic reticulum stress (ERS) that accompanies episodes of T2DM leads to hepatocellular inflammation, injury, hepatic tissue necrosis and severe liver disease, suggesting that T2DM exacerbates the progression of liver disease (48). Nonetheless, our study suggested no causal relationship between T2DM and PBC, but further elucidation of the mechanisms behind the association between these two diseases could contribute to clinical prevention as research progresses.

The primary contribution of our work is that we investigated the bidirectional link between DM and PBC using a 2-sample MR technique for the first time, to the best of our knowledge. This approach is less vulnerable to reverse causality, confounding variables, and exposures that are the same for all groups than observational research. Moreover, the DM subtypes are narrowly defined to remove the impact of coexisting diseases on results. However, the current study has many areas for improvement as well. Firstly, for this Mendelian analysis, we chose the DM subtypes T1DM and T2DM since they had a sample size that was comparatively large enough. Due to the small sample size, gestational diabetes mellitus and diabetes mellitus of specific forms were excluded; these conditions will be further improved after more extensive GWAS data are available. Secondly, despite utilizing the largest GWAS on T1DM, only a limited number of SNPs adhere to genome-wide significance, producing feeble genetic instruments. To address this issue, we relaxed the statistical threshold (*p* < 5 ×10^−6^) to incorporate supplementary SNPs. More research will be required to support our conclusions when more significant GWAS numbers become available. Thirdly, our findings cannot be generalized to other racial groups because the GWAS we used comes from individuals with European ancestry. These constraints require Future research to establish causality and look into probable processes. It is necessary to provide relevant clinical recommendations.

## 5 Conclusions

Our MR analysis indicated a potential causal connection between DM and PBC. However, this association was specific to T1DM and PBC, and no causal link existed between T2DM and PBC. Nevertheless, the available clinical research on the correlation between DM and PBC is currently limited, and comprehensive long-term prospective studies are imperative to enhance our comprehension of the causal relationship between DM and PBC.

## Data availability statement

The datasets presented in this study can be found in online repositories. The names of the repository/repositories and accession number(s) can be found in the article/**Supplementary Material**.

## Author contributions

DL: Writing – original draft, Data curation. HW: Writing – original draft, Methodology. YL: Writing – review & editing, Formal analysis. SC: Writing – original draft, Investigation. HS: Writing – original draft, Visualization. XM: Writing – review & editing, Software. TL: Writing – review & editing, Supervision. ZX: Writing – review & editing, Writing – original draft, Supervision, Formal analysis.
