# Supplementary material for: Association between diabetes mellitus and primary biliary cholangitis: a two-sample Mendelian randomization study
Source: Front Endocrinol (Lausanne). 2024 May 7;15:1362584. doi: 10.3389/fendo.2024.1362584 (PMC11106416; doi:10.3389/fendo.2024.1362584)
Supplement: Supplementary file 1 [file DataSheet_1.docx]

Supplementary Figures


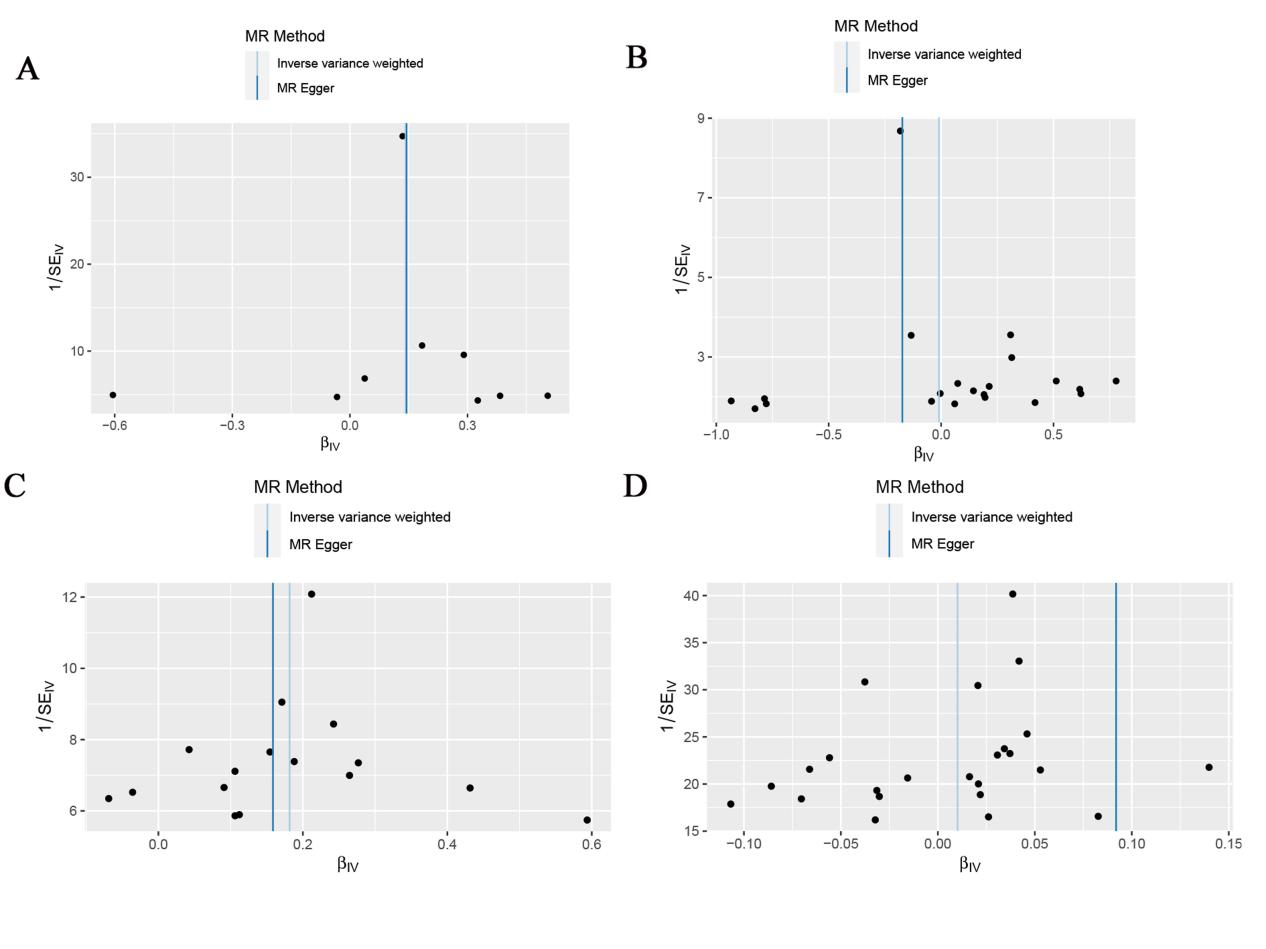


**Supplementary Figure S1.** Funnel plots of primary MR analysis. (A) T1DM on PBC; (B) T2DM on PBC; (C) PBC on T1DM; (D) PBC on T2DM. T1DM, Type 1 diabetes; T2DM, Type 2 diabetes; PBC, primary biliary cholangitis; MR, Mendelian randomization; IVW, inverse variance weighted; WM, weighted median.


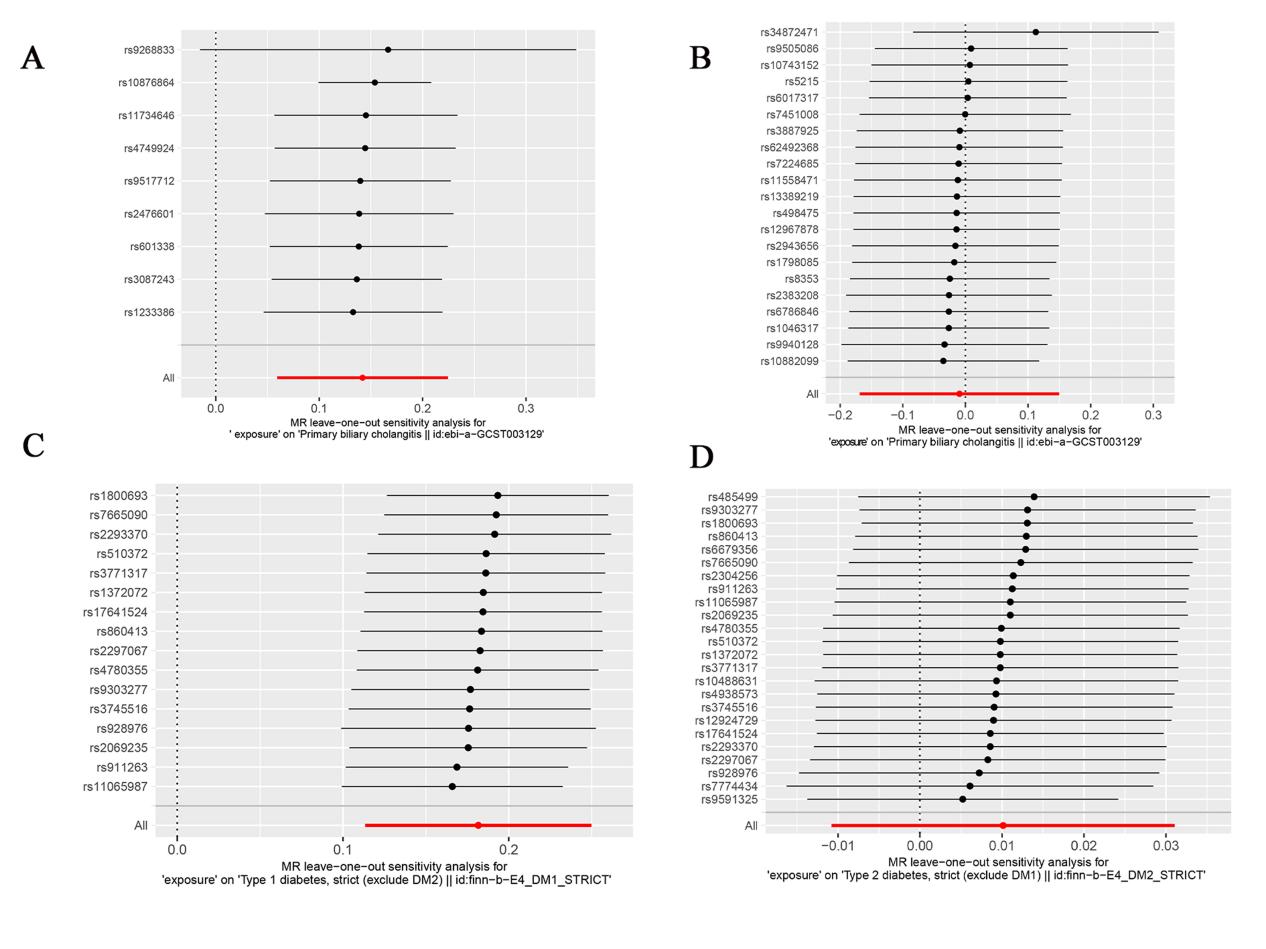


**Supplementary Figure S2.** Leave-one-out plots of primary MR analysis. (A) T1DM on PBC; (B) T2DM on PBC; (C) PBC on T1DM; (D) PBC on T2DM. T1DM, Type 1 diabetes; T2DM, Type 2 diabetes; PBC, primary biliary cholangitis; MR, Mendelian randomization; IVW, inverse variance weighted; WM, weighted median.


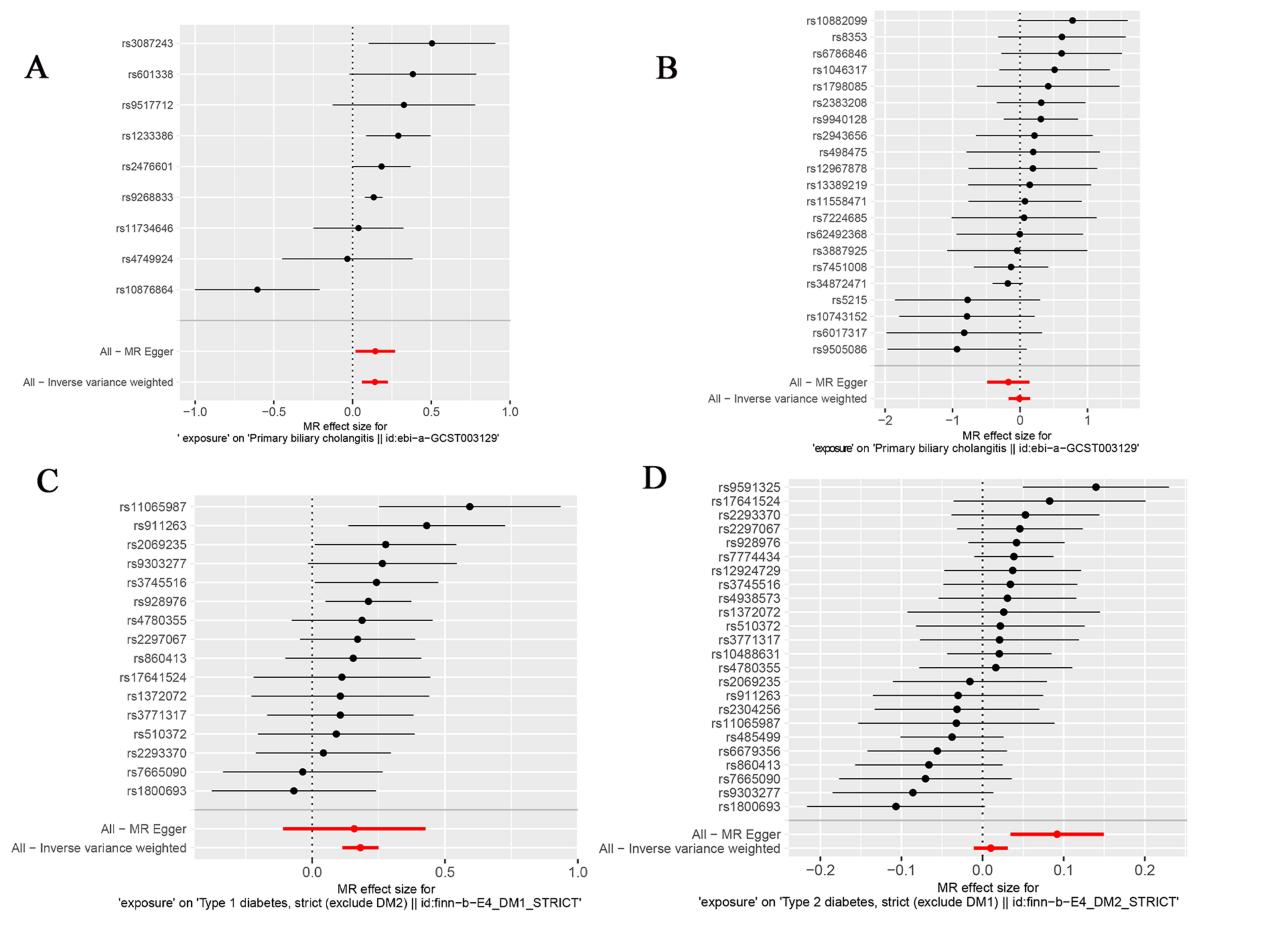


**Supplementary Figure S3**. Forest plots of primary MR analysis. (A) T1DM on PBC; (B) T2DM on PBC; (C) PBC on T1DM; (D) PBC on T2DM. T1DM, Type 1 diabetes; T2DM, Type 2 diabetes; PBC, primary biliary cholangitis; MR, Mendelian randomization; IVW, inverse variance weighted; WM, weighted median.
